# Spatial Frequency Tuning and Transfer of Perceptual Learning for Motion Coherence Reflects the Tuning Properties of Global Motion Processing

**DOI:** 10.3390/vision3030044

**Published:** 2019-09-02

**Authors:** Jordi M. Asher, Vincenzo Romei, Paul B. Hibbard

**Affiliations:** 1 Department of Psychology, University of Essex, Wivenhoe Park, Colchester CO4 3SQ, UK; vincenzo.romei@unibo.it (V.R.); phibbard@essex.ac.uk (P.B.H.); 2 Dipartimento di Psicologia and Centro Studi e Ricerche in Neuroscienze Cognitive, Campus di Cesena, Università di Bologna, 47521 Cesena, Italy

**Keywords:** perceptual learning, transfer, specificity, global motion, frequency tuning, psychophysics, contrast sensitivity, internal feedback, external feedback

## Abstract

Perceptual learning is typically highly specific to the stimuli and task used during training. However, recently, it has been shown that training on global motion can transfer to untrained tasks, reflecting the generalising properties of mechanisms at this level of processing. We investigated (i) if feedback was required for learning in a motion coherence task, (ii) the transfer across the spatial frequency of training on a global motion coherence task and (iii) the transfer of this training to a measure of contrast sensitivity. For our first experiment, two groups, with and without feedback, trained for ten days on a broadband motion coherence task. Results indicated that feedback was a requirement for robust learning. For the second experiment, training consisted of five days of direction discrimination using one of three motion coherence stimuli (where individual elements were comprised of either broadband Gaussian blobs or low- or high-frequency random-dot **Gabor patches**), with trial-by-trial auditory feedback. A pre- and post-training assessment was conducted for each of the three types of global motion coherence conditions and high and low spatial frequency contrast sensitivity (both without feedback). Our training paradigm was successful at eliciting improvement in the trained tasks over the five days. Post-training assessments found evidence of transfer for the motion coherence task exclusively for the group trained on low spatial frequency elements. For the contrast sensitivity tasks, improved performance was observed for low- and high-frequency stimuli, following motion coherence training with broadband stimuli, and for low-frequency stimuli, following low-frequency training. Our findings are consistent with perceptual learning, which depends on the global stage of motion processing in higher cortical areas, which is broadly tuned for spatial frequency, with a preference for low frequencies.

## 1. Introduction

Perceptual learning has attracted much attention as a potential tool to aid recovery of lost visual function for clinical populations [1]. Perceptual training (with or without non-invasive brain stimulation) has also been successfully used with optical defects such as myopia [2,3,4,5], amblyopia [6,7,8], presbyopia [8] and cortical damage [9,10,11]. This demonstrates the potential for sensory plasticity in adulthood and suggests that sensory development is not restricted to a critical period early in life [12,13]. Although it has repeatedly been established that training can improve perceptual abilities [14], these benefits tend to be highly specific for both the perceptual features of the stimuli [15,16,17] and the behavioural task used in training [18]. This specificity limits the effectiveness of perceptual learning as a general therapeutic tool. Resolving the conditions under which learning is tied to the features and tasks used in training, and how much it can generalise to new tasks and stimuli, is imperative for understanding the mechanisms of perceptual learning [14,19]. Our study aims to explore the specificity or otherwise for spatial frequency when learning direction discrimination for a motion coherence task. We do this by evaluating the spatial frequency tuning of improvements in performance for trained and untrained stimuli and tasks.

### 1.1. Specificity

A hallmark finding of early perceptual learning research was its focus on rigid specificity to the dimensions of the stimulus used for training. Specificity of perceptual learning for orientation has been shown for tasks based on simple [16,20,21] and more complex features [22]. Furthermore, specificity has also been found for spatial frequency [14,16,23], direction of motion [18], retinal location [17,21,24,25,26,27] of stimuli and the eye to which they are presented [17,25,26]. On the basis of these specific improvements, it was originally proposed that the underlying brain area responsible for learning was likely to be the primary visual cortex (V1) [17]. The receptive fields of cells in V1 display a similarly high degree of specificity to orientation [28,29], spatial frequency [28,30] and direction of motion [31,32]. The receptive fields in V1 are small and only respond to a limited area of the visual field [33,34,35]. However, perceptual learning occurs for tasks that are more complex than can be solved locally [36,37,38,39], which suggests that learning is not restricted to the initial encoding of information in V1. In contrast, the receptive fields in higher cortical areas, such as those found in V3, V4 and V5, are larger than those of V1, and their responses generalise more over stimulus dimensions, for example being less dependent on the location and retinal size of stimuli, the viewpoint of the observer and the prevailing lighting conditions [30,33,40,41,42,43,44]. The receptive fields of neurons in higher cortical areas integrate and pool information across multiple V1 receptive fields [45,46,47,48,49].

### 1.2. Perceptual Learning and the Visual Hierarchy

The visual hierarchy is not organised in a simple feedforward network, consisting purely of upward projections from lower to higher levels. Rather, while V1 sends most of its feedforward output to V2, there are also direct feedforward connections to V3 and V5 [50]. Furthermore, there are re-entrant feedback connections from higher to lower areas, which are argued to be fundamental to efficient cortical organisation [51]. V1 receives strong feedback projections from V2 [50] and V5 [51]. These feedback connections play an important role in the perception of motion [52,53,54]. The reverse hierarchy theory, a theoretical model of perceptual learning, proposed that learning occurs at higher levels of processing and subsequently progresses backwards to the input levels, through top-down guidance via re-entrant connections [55]. This theory predicts that an increase in sensitivity at lower cortical areas would be as a result of the feedback connections from higher cortical areas. Thus, paradigms that incorporate the higher stage (global) aspects of perception to evaluate the transfer of learning may shed light on the nature of these learning mechanisms. In the case of motion coherence, improvements would for example be predicted to be dependent on the spatial frequency tuning of the higher stage motion detectors [56]. The broader frequency tuning found at this level, in comparison with the local stage of motion processing [46], is characteristic of the generalising properties of higher level processing [55].

Levi et al. [36] found broad generalisation of perceptual learning across tasks. Following training on a higher level motion coherence task, they found post-training improvement for an unrelated contrast sensitivity task [36]. This is a particularly notable result, in that training on a globally-processed task improved contrast detection, which is known to rely on early visual locations such as the 4Cα layer of V1 [23]. There is additional support for global motion coherence training improving sensitivity for detection and discrimination from studies that have shown that this training can help recover some of the blind field for cortically-blind subjects. Huxlin et al. [9] (p. 11) showed post-training improvements for V1 damaged subjects in their “blind field”. A key feature of the improvement was its specificity to the trained location. Huxlin et al. suggested this may be explained by islands of activity along the perimeter of the lesion, being stimulated or reactivated through the feedback connections from higher to lower level visual processing areas. This proposal is consistent with the reverse hierarchy theory [55]. As such, training using high-level coherence tasks may result in general improvements in visual sensitivity [36]. This suggests that global motion coherence is an interesting task with which to investigate transfer.

### 1.3. Low- and High-Level Perception of Motion

The spatial frequency tuning of motion detectors differs according to the level of the visual hierarchy processing the sensory input, in a manner that is consistent with the generalising properties of higher level neurons. At the lowest levels, motion signals are encoded locally within the relatively small receptive fields of V1 neurons [32,47]. However, in order to process more complex motion, ambiguous or conflicting signals from the first stage need to be integrated to provide a global representation of motion [57,58]. This integration requires pooling and summation of the information across spatial position, spatial frequency and orientation [46], such that the receptive fields of higher level neurons are larger and more broadly tuned for spatial frequency and orientation. A number of areas within the visual hierarchy play a functional role in processing motion. Areas V2 and V4 have a role in processing moving orientation signals [59,60]. V3A also plays a role in several aspects of motion processing [40], with 76% of neurons being selective for orientation and 40% showing strong direction selectivity. However, evidence from lesion studies [61,62], extra-cellular recordings [63,64] and neuroimaging in humans [65] support area V5 as a brain area that is heavily involved in processing global motion [61,62,63,64,66,67]. Most neurons in V5 are strongly direction selective [65,68], and the evidence for the role it plays in spatially integrating motion signals is well supported by non-human primate data and neuroimaging studies in humans [33,64,69,70]. Receptive fields in V5 can be up to ten-times larger than those in V1 [43], with broad spatial frequency and orientation tuning, allowing them to sum the responses of V1 neurons across space, orientation and spatial and temporal frequency [35]. Given the differences in spatial and temporal frequency tuning between the early and later stages of processing [28,64,69], measuring the tuning of training for each of these dimensions allows us to understand the role of each level in global motion learning. As hypothesised by the reverse hierarchy theory [56], transfer across features of a task such as spatial frequency would be consistent with the tuning of higher stages of motion processing.

### 1.4. Studying Motion Perception

**Figure 1:**
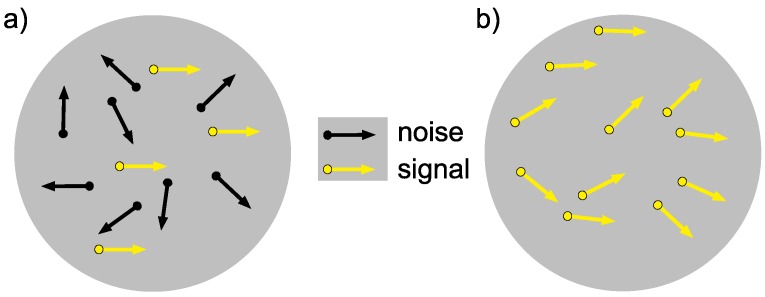
Schematic examples of global motion coherence tasks; both schematics represent dots in motion. An observer would be required to make a left/right response based on his/her perceived direction of motion. Black dots represent noise dots, and yellow dots with a black border represent signal dots. The arrows represent the direction of motion of the dots. (**a**) is a typical stimulus comprised of signal dots and noise dots (approximately 30% coherence). This task requires the observer to segregate and ignore the noise and report the signal direction. (**b**) An equivalent noise stimulus where all dots contribute equally to the signal and the noise (drawn from a distribution of approximately 90° out of a possible 360°). In this task, there are no explicitly designated signal dots, and the direction decision requires the observer to integrate all of the dots to infer the mean direction. In each case, the correct response would be that the dots are moving rightwards.

Global motion coherence is typically studied using random dot kinematograms, requiring observers to make a direction judgement from a stimulus comprised of a pattern of moving dots. A typical stimulus will often contain a proportion of signal dots moving in one direction and noise dots moving in random directions [18,37,71] (Figure 1a). Difficulty is increased by reducing the ratio of signal-to-noise dots; the more noise dots, the lower the coherence and the more difficult the task. In order to perceive a coherent global motion, observers need to segregate the motion signals over space and time [47]. Another method of investigating motion coherence is to use an equivalent noise paradigm [36,72,73,74]. In these tasks, rather than having distinct populations of signal and noise dots, all dots contribute to the signal and the noise by drawing the direction of motion for each dot from a random distribution. Dots move the same distance between each frame, and the direction travelled is independent of the directions of the other dots [74]. Difficulty is increased by manipulating the standard deviation of the distribution of directions presented; thus, each dot contributes to the signal [73] (Figure 1b).

### 1.5. Feedback in Perceptual Learning

Despite the complicated assessment of an ever-changing environment, observers are able to interpret incoming sensory data rapidly, even in novel environments [75]. Furthermore, it is argued that there would be an evolutionary advantage to those organisms who are able to synthesis performance feedback to produce a more efficient learning strategy [76]. Performance feedback is information that notifies a learner about his/her performance and can be generated internally or be provided by an external source [77]. In psychophysical experiments, this often takes the form of an auditory sound that is presented after a trial to indicate a correct or incorrect response. Behavioural perceptual learning studies have shown that using external feedback can improve learning and increase efficiency [78].

However, the role of feedback in perceptual learning is still unclear. Several studies have found that feedback on performance has increased learning [79,80,81] or is a necessary factor [82]. There are also those that find learning occurs without external feedback [83,84,85]. However, feedback is not usually the primary area of interest in perceptual learning studies, the empirical findings cover a pattern of differing methods, stimuli, and feedback usage [82].

In an early attempt to clarify the role of feedback in learning, Herzog and Fahle [79] tested six groups of learners using a vernier acuity task. Feedback was manipulated for each groups as follows. The first group was provided with trial-by-trial feedback, and all observers bar one displayed significant improvement. The second group received no external feedback, and their results were highly variable; there was no significant improvement, most with no change and some who performed worse after training. The third group received end-of-block feedback, provided by percentage correct after each block. Interestingly, improvements were similar to those found for individuals receiving trial-by-trial feedback. The fourth group was provided with random trial-by-trial feedback. There was no learning. The fifth group of observers received partial feedback, on 50% of their incorrect responses, and showed some improvement; however, this was less than those receiving full feedback. The sixth and final group received reverse feedback, and all but one observer adapted.

Herzog and Fahle [79] argued that correct feedback improves both the speed of learning and overall improvement in performance. Furthermore, feedback played a role in reducing the variability in responses over observers. Trial-by- trial feedback was more effective than 50% feedback on incorrect responses. End of block feedback was as effective as trial-by-trial feedback. In the no feedback condition, results were highly variable among observers, and on average, no learning was found without feedback. Random (uncorrelated feedback) was found to prevent learning and thus is as reliable as no feedback at all. The lack of learning without feedback, they argued, suggests that learning from exposure only is implausible. Finally, they suggested that external feedback has an important role to play in learning that cannot be explained (exclusively) as a teaching signal, since block feedback had no signal to individual stimuli, yet learning occurred at the same rate as trial-by-trial feedback [79].

Petrov et al. [86] suggested that feedback may be useful when the stimulus is difficult to detect or discriminate, where it may increase confidence and make learning more efficient. When the stimulus is difficult to detect, external feedback may be useful in increasing observer confidence and increase learning efficiency [86]. Liu et al. [83] predicted that there was an interaction between accuracy and feedback. When accuracy was high for a sufficient number of trials, Hebbian learning predicts a high chance of learning; however, when accuracy is low, Hebbian learning alone is erratic. Alternatively, when feedback (trial-by-trial) is provided, there should be less reliance on performance accuracy. They tested this prediction in a six-day contrast sensitivity (with noise) paradigm using a staircase method. Observers were divided into high and low accuracy training groups, half of whom received trial-by-trial feedback and half of whom did not. They found an interaction between feedback and accuracy; when accuracy was high, external feedback was not critical, but it was crucial when accuracy was low. Furthermore, Liu et al. [83] replicated the study, finding that by mixing high- and low-accuracy trials, learning also occurred without the need for external feedback.

Using two studies, Seitz et al. [82] also investigated if including easy exemplars could foster perceptual learning in the absence of feedback. Firstly, a motion direction discrimination task with low contrast dots and secondly, an orientation discrimination (masked with noise) task using off-cardinal (obliquely) oriented bars. While both groups receiving external reinforcement displayed perceptual learning effects, those that experienced no feedback failed to show learning. They concluded that internal reinforcement was not enough to generate reinforcement signals.

Since the role of feedback is not a routinely tested paradigm, with only a handful of studies that explicitly tested for differences in learning as a result of feedback, the role of external feedback in perceptual learning remains unclear [82] (p. 972).

## 2. Our Study

The purpose of the current study was to determine how the spatial frequency tuning of the neural mechanisms that underpin the perception of coherent motion influenced learning and transfer. Specifically, we wished to establish whether this followed the broad, relatively low-pass tuning properties of the higher stages of motion processing and the degree to which training on this task generalised to a static contrast sensitivity task. This might be expected if global motion training produces general improvements in visual perception [36]. However, prior to collecting the data to understand the spatial frequency tuning of learning for motion coherence, we also questioned whether trial-by-trial feedback was a requirement for learning. Therefore, we first investigated the necessity of feedback for perceptual learning to occur for the specific stimuli used by [9,36].

### 2.1. Experiment 1

The specific nature of feedback and its role in perceptual learning is unclear. While external feedback has been shown to improve learning and increase efficiency [79,81], some studies have found that learning occurs without external feedback [14,18,27,83,85,86]. Recently, it was found that interleaving high-accuracy (easy) trials and low-accuracy (difficult) trials resulted in perceptual learning without the need for feedback, even on difficult trials [83]. Based on these results, we predicted that we should find learning both with and without trial-by-trial feedback, as long as easy and difficult trials were interleaved. As detailed in the following sections, our study found only minimal evidence of learning when no feedback was provided, even with easy trials present. Robust learning only occurred for the feedback condition. With this in mind, our design for Experiment 2 included trial-by-trial feedback during training, but no feedback when testing.

### 2.2. Experiment 2: Main Study

The purpose of this study was firstly to extend the pre- and post-training measures used by Levi et al. [36], to assess the spatial frequency tuning of the mechanisms involved in perceiving and learning motion coherence tasks and, secondly, to assess possible transfer of this learning to a contrast sensitivity task. Improved contrast detection following training on a global motion task has been reported for drifting targets primarily in the low spatial frequency range [36]. However, we questioned how dependent improvement was on the specific temporal and spatial features of the training stimuli. Thus, as well as testing for transfer to static contrast sensitivity tasks, we included measures of transfer to the trained and untrained global motion spatial frequencies. Based on previous findings [36] and the broad frequency tuning of the higher processing stages, we would expect learning and transfer to be particularly strong for low-frequency stimuli. The narrow spatial frequency tuning of the early visual processing stages is well established [46]; thus, transfer between conditions, or a bias towards low spatial frequencies, would support the involvement of higher processing stages, including the re-entrant feedback connections and the reverse hierarchy theory.

## 3. Methods and Materials

### 3.1. Participants

Twenty four observers for Experiment 1 and 30 (new) observers for Experiment 2 were randomly and evenly assigned into groups. For Experiment 1, there was a feedback and a no-feedback group. For Experiment 2, groups were categorised by the spatial frequency of training (broad, low, high). As this is a novel experimental design, expected effects sizes required for precise power analysis calculations were not possible. However, the sample size were chosen so as to be comparable with similar published studies [36,82,83]. All observers were employees or students from the University of Essex and self declared as having normal or corrected-to-normal vision. All work was carried out in accordance with the Code of Ethics of the World Medical Association (Declaration of Helsinki). The study procedures were approved by the University of Essex Ethics Committee (JA1601). All observers gave informed written consent and were either paid or received course credit for their participation.

### 3.2. Experiment 1

**Figure 2:**
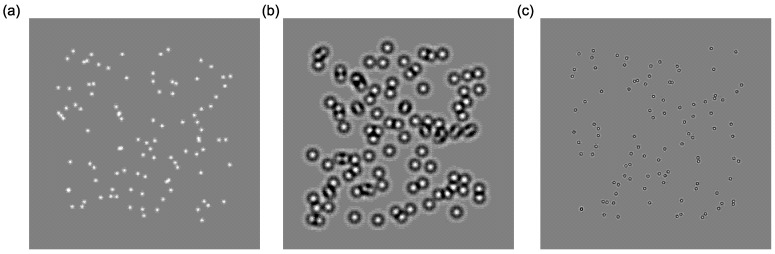
Global motion stimuli, at (**a**) broad, (**b**) low and (**c**) high spatial frequencies. For all stimuli, the spatial frequency of the elements and speed of motion were held constant.

**Figure 3:**
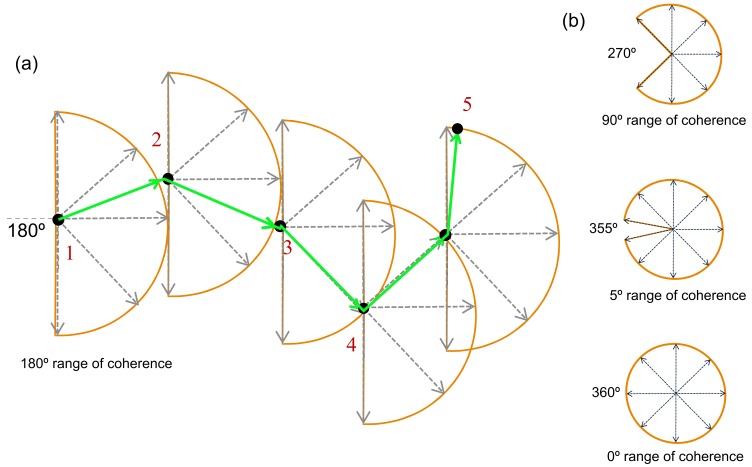
Equivalent noise global motion stimuli. (**a**) The random walk that creates the motion of dots with a 90° arc (for descriptive purposes only). The arc defines the potential area of movement each dot could take at that level. The higher the degree of the arc, the lower the range of potential directions. The arrow indicates the actual trajectory and motion of the dot on each step. (**b**) Potential trajectories for the random walk for levels with 270° 355° and 360° arc.

Stimuli were generated and presented with MATLAB 2015a using the Psychophysics Toolbox extensions [87,88,89]. The broadband motion coherence stimuli were presented using a 2.7-GHz iMac running OSX 1.9.5. The 27″ monitor had a display resolution of 2560 × 1440 pixels and a 60-Hz refresh rate. Viewing distance was 450 mm. The stimuli subtended a visual angle of 66.8°, and one pixel subtended 1.77 arc minutes. Motion stimuli contained 100 Gaussian elements, each with a standard deviation of 6.8 arc minutes (see Figure 2a). Motion stimuli were based on the task designed by Williams and Sekuler [74], where the range of the direction of motion was drawn from a uniform probability distribution, defined by the degree of the angle. The smaller the angle used for the random walk, the fewer direction trajectories are available, which increases the coherence of motion (less random). In contrast, increasing the angle used for the random walk increases the potential trajectories and reduces the coherence (more random). This is illustrated in Figure 3a, which describes the random walk for a single dot with a set level (degree of angle) of 180°, and Figure 3b shows the potential trajectories as the degree of angle increases. Thus, for the purposes of this study, we define coherence in terms of how random the motion appears where the lowest coherence 5° represents motion drawn from a possible distribution of 355°. Levels (coherence) were determined using a pilot to establish an even distribution of easy trials (85% accuracy and above) and difficult trials (65% accuracy and below), with the balance around 75%, as defined by Liu et al. [83]. Accuracy was calculated as the average percentage correct across participants at each level of coherence and is reported in Table 1.

Elements were presented within a mid-grey rectangular region measuring 17.6° × 17.6° on a mid-grey background. Elements were presented for 1 s, moving 5 pixels per frame and a total distance of 8.8°. Dots wrapped around the edges of the rectangle. The seven levels of coherence were: 5°; 10°; 15°; 20°; 25°; 60°; 180°. Trials were presented in a random, interleaved order within each block or session using the method of constant stimuli (MOCS) and requiring a two-alternative-forced-choice (2AFC). There were 40 repetitions of each level, and responses were obtained via the left or right arrow key associated with the perceived direction of coherent motion. Feedback (if present) consisted of an auditory beep immediately following each response, a high-pitched tone for a correct response (2000 Hz for 10 ms) and a low-pitched tone for an incorrect response (200 Hz for 40 ms).

### 3.3. Experiment 2

A series of baseline measures was completed for motion coherence and contrast sensitivity. Test (pre and post) stimuli were presented on a VIEWPixx/3D 23.6-inch monitor with a display resolution of 1920 × 1080 pixels and a 120-Hz refresh rate, using a Dell Precision T3610 PC running Windows 7. One pixel subtended 1.6 arc minutes, and stimuli were viewed for 1000 milliseconds moving 5 pixels per frame (60 frames per second) from a distance of 570 mm. Head position for testing was stabilised using a chin rest. Between these testing sessions, observers undertook five consecutive days of global motion training in one of three spatial frequency groups (broad, low or high). Training stimuli were presented on a 19″ monitor with a display resolution of 1980 × 1080 pixels and a 60-Hz refresh rate, using a PC running Windows 7. Stimuli and step size for the random walk were adjusted to standardise the stimuli across the viewing conditions of the two monitors. One pixel subtended 1.7 arc minutes. Observers were positioned with a viewing distance of 500 mm, which was checked routinely with a measured piece of string. The stimuli were presented for 1000 milliseconds, moving 10 pixels per frame (30 frames per second).

#### 3.3.1. Training Stimuli

*Global motion:* Broadband stimuli were the same as previously described. For low-frequency stimuli, the elements were circularly symmetric Gabor patches. The standard deviation of the Gaussian window, σ, was 30.1 arc minutes, and the spatial frequency of luminance modulation, *f*, was 1 cycle/degree. For each element, the luminance profile was defined as a function of horizontal and vertical position (x,y) as:

(1) $$\begin{array}{c} d\left(x,y\right)=\sqrt{{\left(x-x_{0}\right)}^{2}+{\left(y-y_{0}\right)}^{2}} \end{array}$$

(2) $$\begin{array}{c} L\left(d\right)=\frac{A}{\sigma \sqrt{2\pi }}\exp\frac{-d^{2}}{2\sigma^{2}}\cos2\pi fd \end{array}$$

where $x_{0}$ and $y_{0}$ is the central position of the element and *A* determines its contrast. Elements for the high-frequency stimuli were defined in the same way, but had a standard deviation of 7.48 arc minutes and a spatial frequency, *f*, of 4 cycles/degree. For all stimuli, the spatial frequency of the elements and the speed of motion were held constant. Initially, all elements were uniformly and randomly distributed within a region of 16.6° × 16.6° in the centre of the screen. A central black fixation dot was presented at all times when stimuli were not being displayed. Examples of the stimuli are shown in Figure 2a–c. Motion was created using the method previously detailed, and dots moved a fixed distance of 8.5 arc minutes per frame.

#### 3.3.2. Testing Stimuli (Pre and Post)

**Figure 4:**
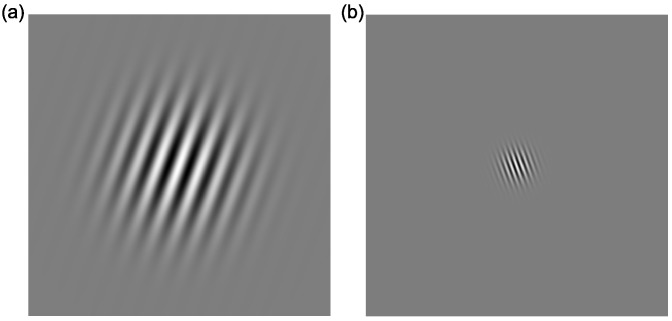
Example of Gabor gratings used for contrast detection for stimuli with (**a**) 1 cycle per degree at +20° (**b**) and 4 cycles per degree at −20°.

1. *Global motion:* Stimuli were identical to those described in the training session, with the following exceptions. In order to standardise the stimuli across the viewing conditions of the two monitors, the standard deviations of the testing elements were 6.4 arc minutes (broadband), 28.4 arc minutes (low-frequency) and 7.0 arc minutes (high-frequency). Stimuli were presented within a mid-grey rectangle measuring 15.9° × 15.9°, and each element moved a fixed distance of 8 arc minutes.
2. *Contrast sensitivity:* Stimuli were static oriented Gabor patches (see Figure 4), with a spatial frequency of 1 cycle per degree (/°) or 4 cycles/°, presented in the centre of the screen on a mid-grey background, tilted either ±20° away from vertical. The Gaussian envelope of the Gabor stimulus had a standard deviation of 1.1°. Seven levels of contrast (0.05, 0.1, 0.15, 0.175, 0.2, 0.3, 0.4% Michelson Contrast) were presented.

### 3.4. Procedure

For each observer, training was undertaken at one spatial frequency only, totalling 420 trials daily for 5 continuous days. Based on the findings from Experiment 1, feedback was provided after each trial. Testing (pre and post) measures were taken for motion coherence (for all frequencies) and contrast sensitivity (high and low spatial frequency). Responses were captured on a DataPixx response box for contrast sensitivity and left and right arrows on the keyboard for the motion task. The presentation order of trials was randomised for direction and coherence (for global motion), spatial frequency and orientation (for contrast sensitivity). There were 20 repetitions for each of the seven levels, for each condition. Testing was performed in a darkened room, before and after training.

### 3.5. Statistical Methodology

Moscatelli et al. [90] proposed using the Generalised Linear Mixed Effects Model (GLMM) for psychophysical data. The GLMM is an extension of the General Linear Model (GLM) and one that provides a more robust statistical analysis where the data contain irregular response distributions. The GLMM contains both fixed and random effects. The fixed component estimates are the effects of interest, for example (a) the day (or session) of testing and (b) each level of the stimulus (coherence, contrast or orientation). The modelling of random effects assesses the differences between related groups (such as those from different observers) that allow inference to a larger population [91]. The strength in the model lies in the model’s flexibility to assign fixed or random parameters for the slope and the intercept.

**Figure 5:**
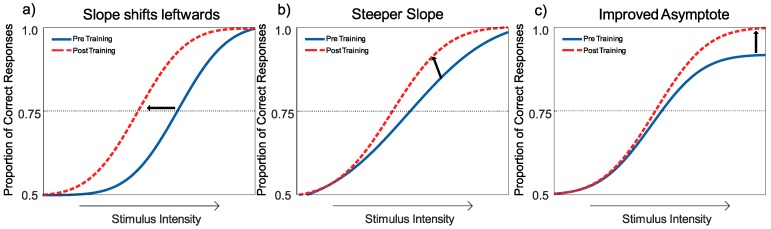
Psychometric functions illustrating the three measures by which the non-linear regression provides evidence of a change. (**a**) A leftward shift of the function indicates a general increase across stimulus levels, which did not vary as stimulus intensity increased. (**b**) A steeper slope indicates an increase in the number of correct responses as stimulus intensity increases. (**c**) An upward shift of the asymptote indicates an increase in performance where stimulus intensity is at its highest. The midpoint is the point where the proportion correct intersects the half-way point between a chance response and perfect (in this schematic, it is 75%). These changes are independent aspects of the psychometric function fit and may not necessarily always be congruent. For example, it is possible to obtain an increase in one measure and a decrease (or no change) in another.

Learning is often measured through monitoring performance at a particular threshold, which is expected to shift the psychometric function leftwards if performance is improved; see Figure 5a. This threshold describes a change in performance (proportion correct) as a function of the strength of the stimulus [92]. The leftward shift in the curve indicates an improvement in threshold (or the midpoint). However, learning may differ across stimulus intensities. For example, an increase in slope indicates an increase in the rate at which performance increases with increasing stimulus intensity (see Figure 5b). The GLMM can fit these two types of learning within its two free parameter model.

However, the GLMM assumes that performance and intensity are positively correlated and reaches asymptotic performance at the highest stimulus intensity [93]. Initial analysis using the GLMM resulted in a poor psychometric fit of the observer response data, where some conditions did reach perfect accuracy, asymptoting at a proportion of correct responses that was less than 1; this resulted in a poor psychometric fit of the observer response data using the GLMM. To accommodate this, a nonlinear generalised mixed effects model (NLME) was used. The NLME includes an additional free parameter (asymptotic performance) to model the variability in responses [91]. An increase in the asymptote indicates a significant change to the performance at the highest level of stimulus intensity (Figure 5c). Allowing the asymptote to vary between observers allows the model to fit responses that do not increase linearly with stimulus intensities. While accounting for asymptotic performance is not new in perceptual learning studies [94,95,96], the NLME fits and analyses the dataset at the level of the sample, rather than the individual. Choosing which free parameters to include in the NLME is complicated, and the strength of any model can only be compared by comparing other simpler fits. We used the guidance from Agresti [91] (and others) by comparing our model to one that only contains the fixed parameter, intercept. Due to the number of analyses we report, we chose to use the same model across all comparisons.

**Figure 6:**
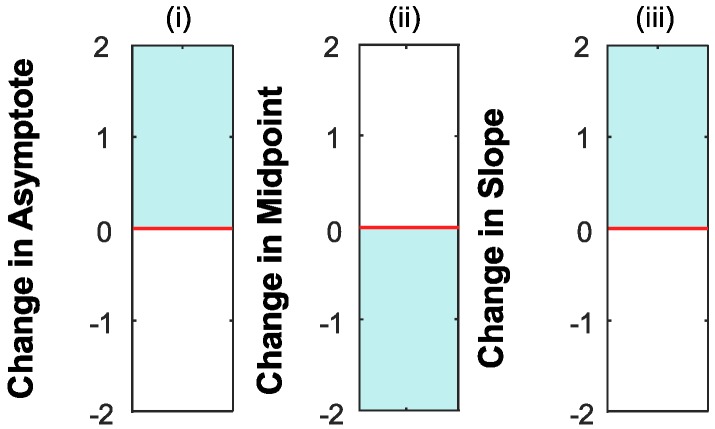
Plots show a visual report of the 95% confidence intervals for the change in (**i**) asymptote, (**ii**) midpoint and (**iii**) slope after training. A positive change to the (**i**) asymptote or (**iii**) slope reflects an improvement in performance, and conversely, a negative change indicates worsened performance. Thus, any results that are exclusively in the shaded areas are indicative of a significant positive change (improvement). Results in the unshaded areas show a significant negative change (worsening performance). Any results that cross both zones (i.e., include zero) show no significant change. In contrast, a positive change to the (**ii**) midpoint (or threshold) shows worsened performance, while a negative change to performance indicates an improvement.

One disadvantage of the NLME is that there is no simple measure of significance provided after the analysis. Significance is thus interpreted using confidence limits, which we have opted to portray visually (see Figure 6i–iii). The 95% confidence intervals that cross (include) zero are not significantly different. Those that are exclusively negative or positive indicate a significant change in that direction.

#### Interpreting the Changes to the Psychometric Function

The change in the upper asymptote can best be interpreted as a change in *response gain*: a change in the maximum response to stimuli as a result of training [94,96,97]. This indicates an increase of performance when the stimulus is at its highest. This is predicted if an increase in weighting occurs over a smaller range of stimulus positions and features (e.g., motion direction) than that used to define the task. The midpoint (in this case 75%) summarises the point at which observers responded correctly on 75% of trials. A reduction in the midpoint is predicted if an increase in the weighting of stimuli occurs over a range of stimulus positions and features greater than that required for the psychophysical task. Finally, a change to the slope parameter shows how the proportion of correct responses changes as a function of stimulus strength.

Perceptual learning was modelled as a linear trend with time, so that we assumed that, if any of these parameters changed as a result of learning, this would be at a constant rate. This linear trend is a first-order approximation; while we would expect any changes as a result of learning to be monotonic, we did not necessarily assume that they would be linear. It should be noted however that clear linear improvements in thresholds were reported by Levi et al. [36]. Since our experiments are closely related to this study, we used a linear model of performance as a simple first approximation.

## 4. Results

### 4.1. Statistical Methods

All analyses were conducted using a Nonlinear Mixed Effects Model (NLME) with MATLAB (using the *nlme* function) and a logistic link function defined by the following model:

(3) $$p=0.5+\frac{\left(A+A_{d}\left(S-1\right)\right)}{1+e^{-\left(K+K_{d}\left(S-1\right)\right)\left(C-\left(C_{0}+C_{0d}\left(S-1\right)\right)\right)}}$$

where *p* is the proportion of correct responses, *A* determines the asymptotic level of performance, *K* defines the slope and $C_{0}$ defines the midpoint of the function. *d* is the post-training change in performance, and $A_{d}$, $K_{d}$ and $C_{0d}$ determine the change in asymptote, slope and midpoint, respectively. *C* is the coherence level, and *S* is the day. Random effects were included for all parameters, and the model was compared to a null model in which only the intercept was free to vary, using the chi-squared statistic to test for a significant improvement in the log likelihood. The 95% confidence limits were calculated for the estimated parameter values. These were plotted for each comparison as a nested bar plot within each model fit (reported below). In the case of the contrast sensitivity task, the model with the full random-effects structure did not converge, and a reduced model with random effects for asymptote and slope, as well as the change in these two parameters between sessions was used instead.

### 4.2. Feedback and Perceptual Learning: Experiment 1

**Figure 7:**
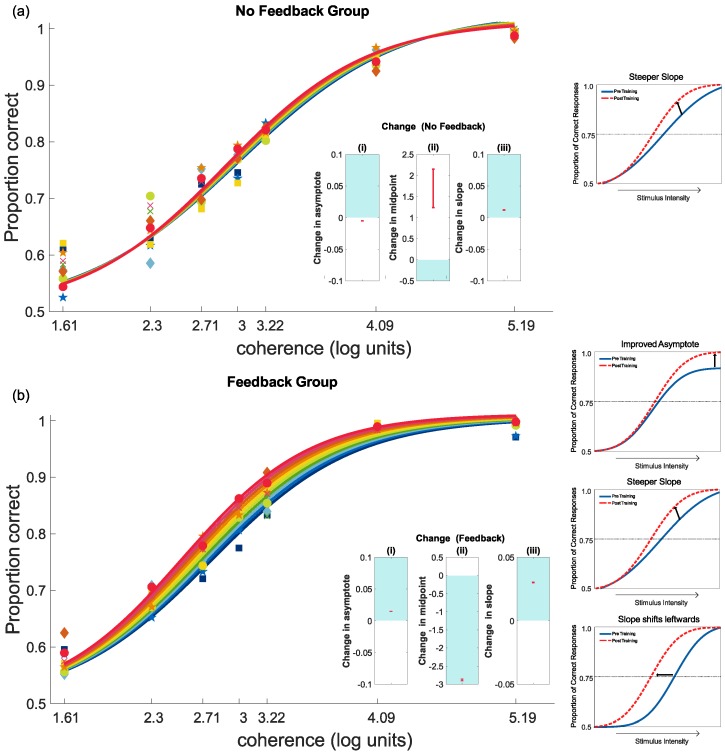
Progress during the ten days of training for each feedback condition scaled to log units for visualisation: (**a**) no feedback and (**b**) with feedback. Nested plots show the 95% confidence intervals for the change for each measure of interest, namely (i) asymptote, (ii) midpoint and (iii) slope. Results that are exclusively in the shaded region indicate a significant improvement in performance. Results wholly in the unshaded areas show a significant worsening in performance. Results that cross both zones (i.e., include zero) show no significant effect of training on that parameter. The mini plots on the far right illustrate the improvement.

Results for the feedback and no-feedback conditions are plotted in Figure 7. To identify if either trial-by-trial feedback or a combination of easy and difficult trials in the absence of feedback produced learning, the response data for each condition (feedback and no feedback) were analysed independently. The seven levels of coherence (5°, 10°, 15°, 20°, 25°, 60°, 180°) were transformed to show the natural log (coherence), to better show the results at low levels of coherence.

For the no-feedback group, there was a small reduction in the asymptote, a significant increase in midpoint and an increase in slope as a result of training. However, the change by day was not significant (Figure 7). In contrast, for the feedback group, there was a significant decrease in midpoint and an increase in slope by day. There was a significant change by day to the asymptote; however, this was a small change. The model fits are summarised in Table 2. All three of these effects indicated an improvement in performance through training. Overall, there was consistent improvement in performance with feedback, but little change when it was absent. These results demonstrate that, for our training procedures, feedback was necessary for learning. Trial-by-trial feedback was thus provided in all training conditions in the second experiment.

### 4.3. Feedback and Perceptual Learning: Experiment 2

#### 4.3.1. Training Results

**Figure 8:**
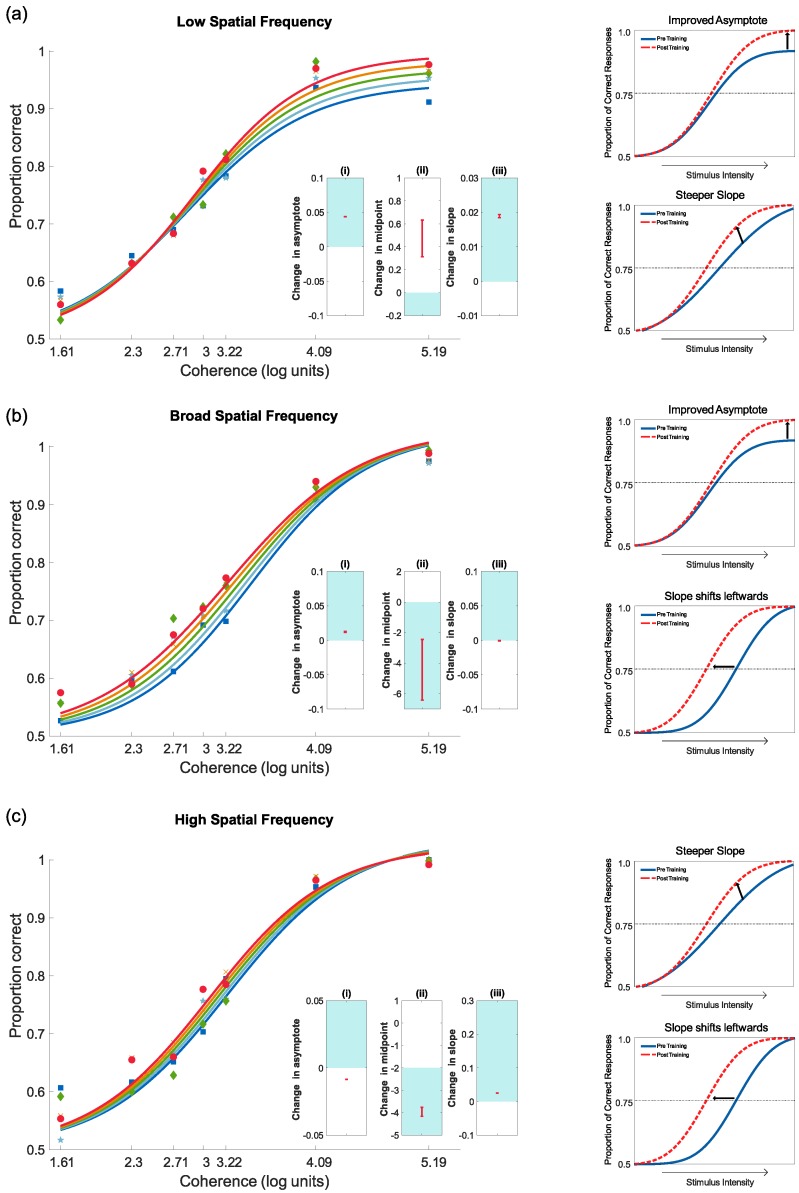
Progress during the five days of training for each frequency trained group scaled to log units for visualisation: (**a**) low, (**b**) broad and (**c**) high. Nested plots show the 95% confidence intervals for the change for each measure of interest. Namely (i) asymptote, (ii) midpoint and (iii) slope. Any results that are exclusively in the shaded area indicate a significant positive change (improvement). Results in the unshaded areas show a significant negative change (worsening performance). Finally, results that cross both zones (i.e., include zero) show no significant difference. The mini plots on the far right illustrate the improvement.

Results for the five days of training, with feedback, are shown in Figure 8. For the group trained with low-frequency stimuli, there was an increase in asymptote, an increase in midpoint, and an increase in slope. For the group trained with broad frequency stimuli, there was an increase in asymptote, a reduction in midpoint, and a change in slope. For the group trained with high-frequency stimuli, there was a reduction in asymptote, a reduction in midpoint, and an increase in slope. As can be seen in Figure 8, performance on the motion coherence task improved across the training session for all three groups, showing perceptual learning for all spatial frequencies. The model fits are summarised in Table 3.

#### 4.3.2. Pre- and Post-Test Results for Motion Coherence

*(a) Low-Frequency Training:*

**Figure 9:**
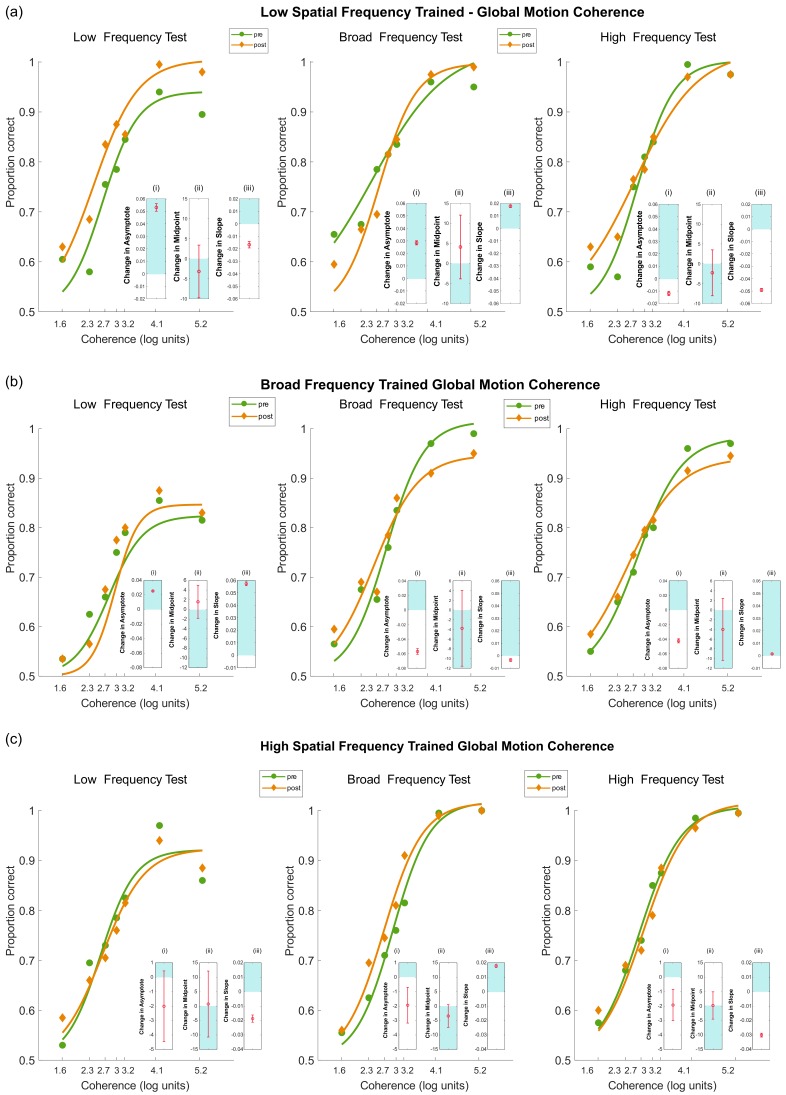
Pre- and post-test motion coherence results following after the five days of training for each frequency trained group scaled to log units for visualisation: (**a**) low, (**b**) broad and (**c**) high. Nested plots show the 95% confidence intervals for the change for each measure of interest, namely (i) asymptote, (ii) midpoint and (iii) slope. Any results that are exclusively in the shaded are indicate a significant positive change (improvement). Results in the unshaded areas show a significant negative change (worsening performance). Finally, results that cross both zones (i.e., include zero) show no significant difference.

Results for the group trained with low-frequency stimuli are shown in Figure 9a. The model fits are summarised in Table 4. When tested with low-frequency stimuli, there was an increase in asymptote and a reduction in midpoint, but a reduction in slope. Overall, the results showed an improvement in performance, particularly at high coherence levels. When tested with broad frequency stimuli, there was an increase in asymptote and an increase in slope, but no change in midpoint. Again, these results showed an improvement in performance, most notably at high coherence levels. Finally, when tested with high-frequency stimuli, there was a reduction in asymptote, a reduction in slope and no change in midpoint. There was no improvement in the perception of motion coherence in this case, rather a slight reduction in performance.

*(b) Broad Frequency Training:*

Results for the group trained with broad frequency stimuli are shown in Figure 9b. The model fits are summarised in Table 4. When tested with low-frequency stimuli, there was an increase in slope and asymptote, but no change in midpoint. As can be seen in Figure 9b, performance improved following training for low-contrast test stimuli in the way of training. When tests had broad frequency stimuli, there was a reduction in asymptote, no change in midpoint and an increase in slope. Unexpectedly, these results showed a reduction in performance at high coherence levels. Finally, when tested with high-frequency stimuli, there was a reduction in asymptote, an increase in slope and no change in midpoint. Again, these results showed an unexpected reduction in performance at high coherence levels after training.

*(c) High-Frequency Training*

Results for the group trained with high-frequency stimuli are shown in Figure 9c. The model fits are summarised in Table 4. Performance on the low-frequency stimuli test showed a reduction in slope, but no change in asymptote or midpoint. There was little evidence for any change in performance following training. When tested with broad-frequency stimuli, there was a reduction in asymptote, no change in midpoint and an increase in slope. Again, there was little evidence for any change in performance following training. Finally, when tested with high-frequency stimuli, there was a reduction in asymptote and slope, but no change in midpoint. As with the other two test frequencies, there was little evidence for any change in performance.

#### 4.3.3. Pre- and Post-Test Results for Contrast Sensitivity

*(a) Low-Frequency Training:*

**Figure 10:**
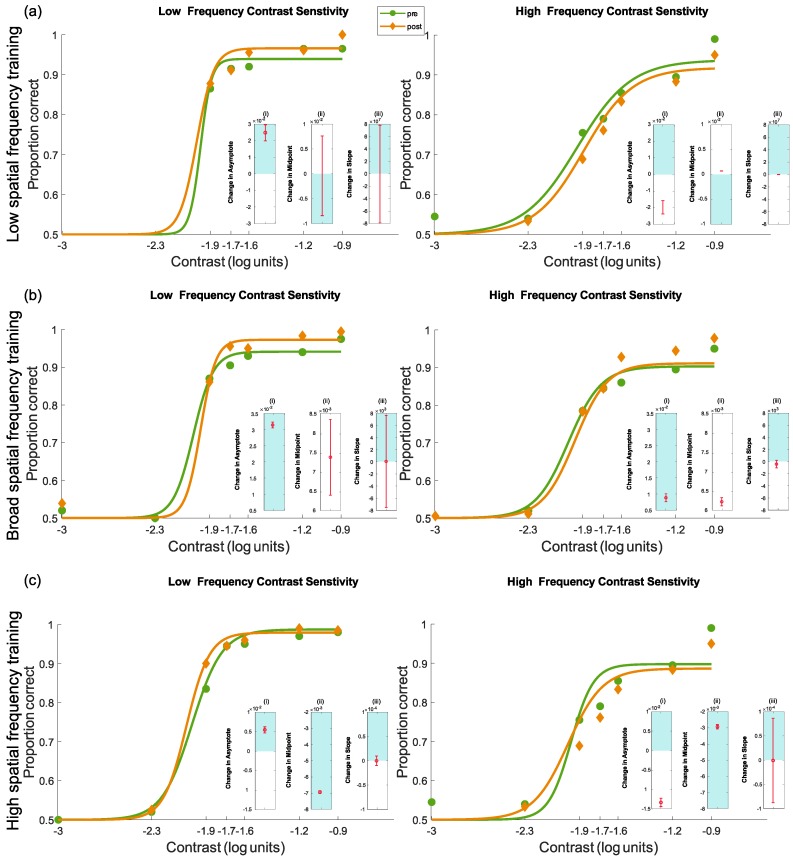
Pre- and post-test contrast sensitivity (low and high spatial frequency) results following the five days of training for each frequency trained group scaled to log units for visualisation: (**a**) low, (**b**) broad and (**c**) high. Nested plots show the 95% confidence intervals for the change for each measure of interest, namely (i) asymptote, (ii) midpoint and (iii) slope. Any results that are exclusively in the shaded area indicate a significant positive change (improvement). Results in the unshaded areas show a significant negative change (worsening of performance). Finally, results that cross both zones (i.e., include zero) show no significant difference.

Results for the group trained with low-frequency stimuli are shown in Figure 10a. The model fits are summarised in Table 5. When tested with low-frequency stimuli, there was an increase in asymptote and a reduction in midpoint, but no change in slope. Overall, the results showed an improvement in performance, particularly at high coherence levels. When tested with high-frequency stimuli, there was a reduction in asymptote, a small increase in midpoint and no change in slope. These results reflected an overall worsening of performance. 

*(b) Broad Frequency Training:*

Results for the group trained with broad frequency stimuli are shown in Figure 10b. The model fits are summarised in Table 5. When tested with low-frequency stimuli, there was an increase in asymptote and an increase in midpoint, but no change in slope. Improvement was evidenced at high stimulus intensities only. When tested with high-frequency stimuli, there was a very small increase in asymptotic performance, an increase in midpoint and no change in slope. These results again reflect an improvement in performance as a result of training, particularly at the higher contrast levels.

*(c) High-Frequency Training:*

Results for the group trained with high-frequency stimuli are shown in Figure 10c. The model fits are summarised in Table 5. When tested with low-frequency stimuli, there was an increase in asymptote and a change in midpoint, but no change in slope. These results reflect a very modest improvement in performance following training. When tested with high-frequency stimuli, there was a reduction in asymptote and a reduction in midpoint, but no change in slope. These results represent a slight worsening of performance following training.

## 5. Discussion

The first experiment assessed the need for trial-by-trial feedback for perceptual learning in a motion coherence task. We found robust learning only when feedback was provided. These results contrast with other studies, in which learning was found to occur without feedback, provided that a combination of both easy and difficult trials was presented [83]. “Easy” trials were defined as those that could be discriminated with 85% accuracy and “difficult” trials those that could be discriminated with 65% accuracy. While the overall accuracy level in our study was very similar, our procedure differed in that we used the method of constant stimuli, so that we presented stimuli at seven different levels, rather than just two. This may have made it harder to generate accurate metacognitive judgements of confidence in perceptual performance [98], which in turn would have adversely affected the appropriate weighting of trials in guiding perceptual learning [99]. These results suggest that the inclusion of easy and difficult trials is not sufficient to ensure robust perceptual learning. Rather, it appears necessary that easy and difficult trials can also be readily discriminated. This would facilitate an efficient, adaptive approach to reweighting. Petrov et al. [86] argued that observers can use their own decision as the training signal to support Hebbian-like learning; if this is correct more often than not, it can be used to guide reweighting in order to improve performance. Learning can be improved by selectively reweighting those trials that can be identified as easy and therefore more likely to support accurate perceptual decisions [99]. When multiple levels of difficulty are presented within an experiment, observers tend to be overconfident in difficult trials and under-confident in easy trials [98]. Such an inaccuracy in meta-perceptual judgements might explain the lack of perceptual learning without feedback in our experiment. These results demonstrate the need for trial-by-trial feedback for robust learning for our stimuli and task.

In the second experiment, participants were tested on all motion coherence conditions (for low-, broad and high-frequency stimuli) and contrast sensitivity (low- and high-frequency Gabor patches). They were then trained on a motion coherence task, with either low-, broad or high-frequency elements, before retesting on all tasks.

### Main Findings

When trained with *low-frequency stimuli*, there was improvement for the low- and broad frequency conditions, but not the high spatial frequency condition. This pattern of learning is consistent with the broad, but relatively low-frequency tuning properties of global motion detectors.

For those trained using *broad frequency stimuli*, improvement at the testing stage was restricted to the untrained low-frequency condition. Based on the frequency tuning properties of global motion detectors, we would also have predicted a similar pattern of learning for broadband test stimuli. With learning, nonetheless driven by the low-frequency content of the stimuli

Finally, when trained on *high-frequency stimuli*, the relatively weak responses of global motion mechanisms to high spatial frequency stimuli may account for the general absence of improvement in performance across all frequencies at the test stage.

When testing for transfer of learning to the contrast sensitivity task, an improvement was found for both low- and high-frequency contrast stimuli following training with *broad frequency stimuli*. Following training with *low-frequency stimuli,* there was an improvement on low-frequency contrast stimuli. These results are again broadly consistent with the spatial frequency tuning of global motion processing, but with broader transfer effects than for improvements in motion. Thus, the small improvement in asymptotic performance in contrast sensitivity for both low and high frequencies following training with broad frequency stimuli may also reflect the frequency-tuned mechanisms that respond to these stimuli, albeit to a lesser extent than for low-frequency stimuli. In contrast, the lack of improvement for high-frequency stimuli is likely to reflect the fact that these narrowband stimuli will not have created sufficiently strong responses in global mechanisms to generate learning. These results are consistent with those of Levi et al. [36], who noted that the broad improvement in contrast sensitivity following training with random dot stimuli reflects the broadband content of these stimuli. We have shown that this learning can be more restricted when training with narrowband stimuli and that transfer did not require the test and training stimuli to have the same temporal properties.

We found improvements in performance on our motion coherence task, following training with trial-by-trial feedback, for all spatial frequencies. These improvements did not in all cases transfer to our post-training testing, without feedback, even when testing with the same stimuli as used for training. In related experiments [36], post-training assessment without feedback was not investigated, so it is not possible to directly compare this aspect of our results. Herzog and Fahle [79] found that, when trained on a vernier acuity task, improvements in performance from training with feedback persisted when feedback was removed. In that study, a single level of stimulus was presented, set to be just below each participants’ midpoint prior to training. One possible difference in our study was that the combination of multiple stimulus levels and the removal of feedback would have adversely affected participants’ metacognitive confidence judgements, which may in turn have lowered their performance on the task [98]. While a full investigation of this is beyond the scope of this project, we can conclude that the strongest and most robust learning occurred following training and testing with the stimuli comprised of low-frequency elements. This is consistent with the spatial frequency tuning of global motion processing.

Overall, we tended to find improvements in performance characterised by a changing asymptote, rather than a reduction in the midpoint of the psychometric function. These results suggest that, in this case, learning acts to increase the response gain, rather than contrast gain. In our version of the global motion task, pooling over the whole of the stimulus, and the full range of directions, will contribute to task performance. The change in asymptote is therefore consistent with a change in input weighting over a more narrowly-defined range of stimulus positions or directions and, thus, a partial, but incomplete, recruitment of the information potentially available.

The spatial frequency tuning of the transfer of learning suggests an important role for the higher level, global stage of motion processing. This result is consistent with reverse hierarchy theory [55], in which learning operates through top-down guidance via re-entrant connections. A critical role for these top-down connections between the global and local stages of motion has been shown through studies using transcranial magnetic stimulation (TMS), which has shown that the feedback connections from V5 to V1 are adaptable [54]. TMS applied asynchronously to V1 and V5 was to enhance the perception of coherent motion, such that thresholds were lower after TMS application. Critically, this was dependent on the timing and direction of stimulation. Motion perception was found to be strengthened when TMS was applied first to V5 then to V1, strengthening the re-entrant connection from V5 to V1, but not when applied first to V1 then to V5 or when stimulation was simultaneous. This asymmetry shows that re-entrant connections play a role in perceptual learning in global motion coherence tasks. Chiappini et al. [100] used a similar paired cortico-cortical paired TMS protocol (ccPAS) to induce a direction-selective improvement in performance by combining sub-threshold stimulation with the simultaneous presentation of direction-specific moving stimuli. This provides accumulating evidence that the re-entrant connections from direction-tuned neurons play a role in perceptual learning in global motion coherence tasks. These results are also consistent with the effects of high-frequency transcranial random noise stimulation (hf-tRNS) in improving perceptual learning in an orientation discrimination task [101]. hf-tRNS has also been shown to improve visual acuity, but not contrast sensitivity, in amblyopia [102,103]. The facilitation of learning by hf-tRNS is believed to reflect the repeated sub-threshold stimulation of cortical neurons [101].

## 6. Conclusions

We assessed the transfer of learning from training on a motion coherence task across spatial frequency and to a static contrast sensitivity task. The transfer of learning reflects the spatial frequency tuning of global motion mechanisms, which are tuned to relatively low spatial frequencies [46]. These results demonstrate the important role played by these global mechanisms in perceptual learning, as predicted by the reverse hierarchy theory [55]. Consistent with this theory, the transfer of learning to a static contrast sensitivity task is likely to reflect the influence of changes from higher stage feedback connections to earlier stages of visual processing.

## Figures and Tables

**Table 1:** Average accuracy across participants for direction detection of motion coherence.

Coherence °	Accuracy (%)
180	98.1
60	96.0
25	80.4
20	77.7
15	73.5
10	64.1
5	61.5

**Table 2:** Goodness of fit tests for each model. A mixed-effects model with the intercept as the only free parameter was compared with the model including the full psychometric function. The −2× difference in log likelihood between the full and null model was calculated and tested for significance against a chi-squared distribution, with degrees of freedom equal to the difference in the number of parameters for the two models.

Feedback Condition	−2LL	ΔDOF	*p*
Feedback	1528.96	10	<0.0001
No Feedback	1428.24	10	<0.0001

**Table 3:** Goodness of fit tests for each model. A mixed-effects model with the intercept as the only free parameter was compared with the model including the full psychometric function. The −2× difference in log likelihood between the full and null model was calculated and tested for significance against a chi-squared distribution, with degrees of freedom equal to the difference in the number of parameters for the two models.

Training Frequency	−2LL	ΔDOF	*p*
Low	641.53	10	<0.0001
Broad	618.32	10	<0.0001
High	765.47	10	<0.0001

**Table 4:** Motion coherence: goodness of fit tests for each model. A mixed-effects model with the intercept as the only free parameter was compared with the model including the full psychometric function. The −2× difference in log likelihood between the full and null model was calculated and tested for significance against a chi-squared distribution, with degrees of freedom equal to the difference in the number of parameters for the two models.

Training Frequency	Tested Frequency	−2LL	ΔDOF	*p*
Low	Low	123.29	10	<0.0001
Low	Broad	146.17	10	<0.0001
Low	High	176.96	10	<0.0001
Broad	Low	161.96	10	<0.0001
Broad	Broad	158.93	10	<0.0001
Broad	High	168.91	10	<0.0001
High	Low	98.04	10	<0.0001
High	Broad	207.56	10	<0.0001
High	High	197.53	10	<0.0001

**Table 5:** Contrast sensitivity: goodness of fit tests for each model. A mixed-effects model with the intercept as the only free parameter was compared with the model including the full psychometric function. The −2× difference in log likelihood between the full and null model was calculated, and tested for significance against a chi-squared distribution, with degrees of freedom equal to the difference in the number of parameters for the two models.

Training Frequency	Tested Frequency	−2LL	ΔDOF	*p*
Low	Low	234.87	8	<0.0001
Low	High	161.14	8	<0.0001
Broad	Low	268.60	8	<0.0001
Broad	High	206.31	8	<0.0001
High	Low	276.63	8	<0.0001
High	High	209.01	8	<0.0001
